# The Biomechanical Importance of Bone Block Positioning in Glenoid Augmentation: Every Millimeter Matters

**DOI:** 10.1177/03635465251322796

**Published:** 2025-03-02

**Authors:** Sebastian Oenning, Jens Wermers, Alina Köhler, Julia Sußiek, Mats Wiethölter, Michael J. Raschke, J. Christoph Katthagen

**Affiliations:** † Department of Trauma, Hand and Reconstructive Surgery, University Hospital Muenster, Muenster, Germany; ‡ Faculty of Engineering Physics, FH Muenster, Muenster, Germany; Investigation performed at University Hospital Muenster, Muenster, Germany

**Keywords:** shoulder instability, glenoid concavity, glenoid bone loss, bone block, Latarjet, glenoid augmentation, biomechanics

## Abstract

**Background:**

In the presence of anterior glenoid bone loss (aGBL), options for bony glenoid augmentation include Latarjet procedures and free bone block transfers. Bone graft placement is challenging, and malposition causes complications, such as recurrent instability or osteoarthritis.

**Hypothesis:**

With minimal changes in bone block positioning, osteochondral shoulder stability cannot be restored sufficiently.

**Study Design:**

Controlled laboratory study.

**Methods:**

In a robotic test setup, 14 human cadaveric scapulae were included. Soft tissue was resected, and matching artificial humeri were selected for each specimen. Testing was performed in 60° of glenohumeral abduction with 50 N of glenohumeral compression and anterior-directed translational force to the humerus. Application of 20% aGBL and screw fixation of artificial bone blocks (artBBs) with different buildup shells allowed the following testing stages: (1) intact, (2) 20% aGBL, (3) flush artBB, (4) 1-mm medialized artBB, and (5) 1-mm lateralized artBB. The stability ratio (SR) and medial-lateral humeral head starting position were assessed.

**Results:**

Specimens with 20% aGBL provided lower mean SRs than native joints (20.6% [SD, 4.7%] vs 27.8% [SD, 6.7%]; *P* < .0001). Flush artBB placement (mean, 35.4%; SD, 7.7%) led to an increased SR compared with both native joints (*P* = .002) and 20% aGBL (*P* < .0001). The mean SR in 1-mm medialized artBBs (21.5%; SD, 5.7%) did not differ compared with that for 20% aGBL (*P* = .908). One-millimeter lateralized artBBs (mean, 40.8%; SD, 5%) provided higher SR and more lateral humeral head starting positions compared with flush artBB (*P* = .003 and *P* = .003, respectively).

**Conclusion:**

In the presence of aGBL, flush bone block placement restores osteochondral glenohumeral stability, while a 1-mm medialized bone block fails to increase stability. Bone block lateralization of 1 mm provides higher stability but is associated with humeral head lateralization.

**Clinical Relevance:**

Glenoid bone block augmentations are established in patients with glenohumeral instability and aGBL. In the case of bone block malposition, complications like recurrent instability or the development of osteoarthritis can occur. This study underlines the importance of accurate bone block placement since only minimum bone block malposition relevantly affects osteochondral shoulder biomechanics.

In the presence of bony Bankart lesions, the amount of critical anterior glenoid bone loss (aGBL) indicating glenoid reconstruction ranges from 13.4% to >20%.^15,36,37,43^ In accordance with the principle of concavity-compression, recent biomechanical studies have demonstrated that the loss of glenoid concavity is crucial for the development of glenohumeral instability.^27,29,41^

For bony glenoid reconstruction in patients with recurrent anterior instability, several techniques have been described. The Latarjet procedure is a well-known option, including the transfer of the coracoid tip and the conjoint tendons to the anterior glenoid.^17,19^ Biomechanically, the conjoint tendons and the subscapularis muscle provide a sling effect, serving as an important stabilizing factor reducing anterior translation of the humeral head.^9,40,44^ Other techniques can be summarized as free bone blocks. Especially autologous, J-shaped bone blocks derived from the iliac crest are commonly used.^2,4,34^ Less frequent options include distal clavicle or distal tibia bone grafts as well as allografts.^6,8,39^

Wellmann et al^40^ described the Latarjet technique to be biomechanically superior to free bone blocks. However, both techniques do not differ regarding the long-term functional outcome, return-to-sport rates, and postoperative complications, including recurrent episodes of shoulder instability.^8,28^

A demanding but yet crucial surgical step during any type of glenoid augmentation is the correct bone block placement. In the literature, the occurrence of medialized graft position after glenoid augmentation differs widely from <10%^1,3,16,20,24,32,38^ to 14.6%^35^ and 19.4%.^18^ Rates of lateralized bone blocks analogically differ from 0% to 53% in various studies.^§^ Hovelius et al^12^ described graft migration >0.5 mm in 5% of all patients. While lateralized bone blocks provide high glenohumeral stability,^23^ at the same time, glenohumeral contact pressure rises, correlating with the development of osteoarthritis.^18,23,24^ In finite element models, Martins et al^23^ described that with a 3-mm lateralized bone block, contact pressures exceed the articular cartilage failure stress during anterior humeral head translation. In contrast, a graft that is too medialized is associated with reduced glenohumeral stability.^12,24^ Hovelius et al^12^ found that 83% of patients with graft medialization >1 cm experienced recurrent glenohumeral instability.

In this biomechanical study, we aimed to clarify the effect of bone block positioning on glenohumeral stability to allow more specific recommendations regarding intraoperative bone block placement. We hypothesized that even with a minimal medialization of the bone graft, glenoid augmentation can fail to restore anterior shoulder stability.

## Methods

### Ethics Approval

All donors of human cadaveric specimens provided written consent for the use of their bodies for scientific and/or educational purposes. The study was approved by the institutional review board (IRB No. 2022-323-f-S, University of Muenster, Muenster, Germany).

### Specimen Preparation

For this study, 14 fresh-frozen human cadaveric scapulae (10 left, 4 right; 8 female, 6 male) were obtained from the University of Lübeck, Germany. The mean donor age was 79 years (SD, 9.8 years). After thawing each specimen at room temperature, the soft tissue was removed, including the labrum, enabling the analysis of the osteochondral, concave-shaped glenoids. Also, the coracoid process as well as the acromion was resected, avoiding deflection of the humeral head during simulated anterior translation.

The scapulae were potted in polyurethane casting resin (RenCast PU; Gößl & Pfaff). To provide reliable accuracy, an individual coordinate system was set for each glenoid. The superior-inferior axis was defined by the line connecting the most cranial and most caudal points of the glenoid, respectively. The anterior-posterior axis was set perpendicularly to the superior-inferior axis and positioned at the maximum anterior-posterior glenoid diameter. The glenoid plane was aligned perpendicular to the medial-lateral axis, which was defined orthogonal to the superior-inferior and anterior-posterior axes. Artificial humeri (1028/1028-20; Sawbones) were potted and equipped with stemless shoulder implants. Using the associated trial heads, an experienced surgeon (J.C.K.) and engineer (J.W.) chose the correct humeral head size for each cadaveric glenoid. In a best-fit approach, the smallest possible humeral head that still completely covered the glenoid cavity was selected. The implants allowed for a standardized humeral articular surface, so that biomechanical testing was not biased by humeral osteochondral lesions.

**Figure 1.**
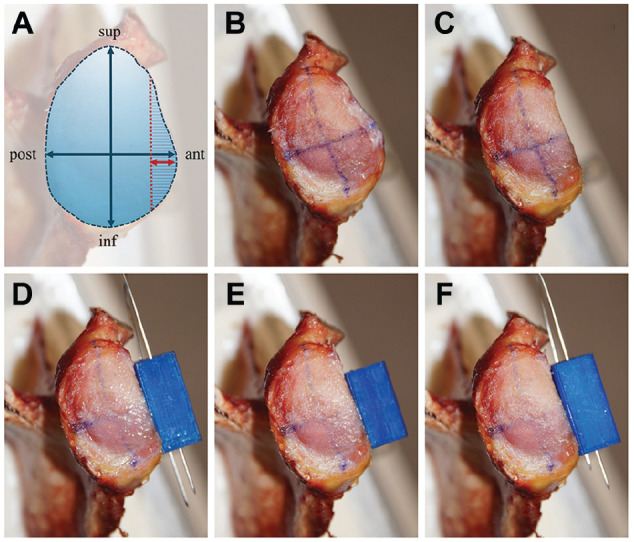
(A) The measurement of 20% anterior glenoid bone loss (aGBL) is outlined. The anterior 20% of the anterior-posterior diameter (red arrow) was marked, and a line parallel to the superior-inferior axis (red dotted line) was drawn to define the area of 20% aGBL (red hatched area). The following images show the 5 different testing stages: (B) intact glenoids, (C) 20% aGBL, (D) an artificial bone block (artBB) in level with the glenoid articular surface, (E) a 1-mm medialized artBB, and (F) a 1-mm lateralized artBB. ant, anterior; inf, inferior; post, posterior; sup, superior.

After testing the intact glenoids as described below, a bony glenoid lesion was applied to each specimen with a hand-guided rotary tool (Multitool 4000; Dremel) (performed by J.W.). Thereby, the anterior 20% of the anterior-posterior axis was marked and a line parallel to the superior-inferior axis defined the area of 20% aGBL, which was then removed (Figure 1). This preparation was based on the work of Saito et al,^33^ analyzing the morphology of aGBL after anterior-inferior shoulder dislocation. Glenoid bone loss was mainly found anteriorly and parallel to the glenoids’ superior-inferior axis at a mean glenoid position of 3:01 (right shoulder).

**Figure 2.**
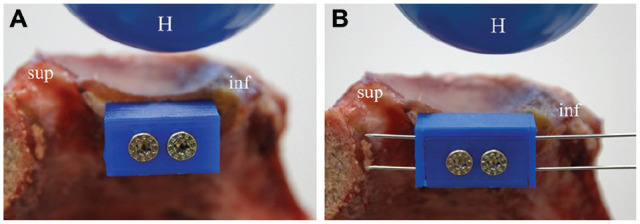
(A) Fixation of the artificial bone block (artBB) to a specimen with 20% anterior glenoid bone loss with two 4-mm screws in a 1-mm medialized position. (B) A 2-mm buildup shell is shown, fixed to the artBB with 2 K-wires, resulting in a 1-mm lateralized bone block position. The superior (sup) and inferior (inf) orientation is shown as well as the artificial humeral head (H).

For bone block preparation, the most physiological proportions of a coracoid transfer were desired to be achieved. With a 3-dimensional (3D) printer (FlashForge Creator Pro 2), polyactic acid blocks of 12 × 12 × 25 mm were produced, as well as 2 additional shells, allowing bone block extension of 1 mm and 2 mm, respectively. The dimensions were based on the work of Montgomery et al,^25^ describing the mean size of coracoid bone blocks in a study including 20 cadaveric shoulders. The artificial bone block (artBB) was fixed to the glenoid by two 4-mm screws. It was positioned parallel to the glenoid superior-inferior axis at the area of aGBL. To facilitate the desired testing stages, the artBB combined with the 1-mm shell was fixed to the anterior glenoid, resulting in a plane articular surface without any gap. To simulate a 1-mm medialized bone block, the 1-mm shell was removed. After that, fixing the 2-mm shell to the artBB simulated a lateralized bone block with a 1-mm gap within the glenoid articular surface. The shells were fixed to the artificial bone block with K-wires (Figure 2).

### Test Setup

Anterior translation of the artificial humeri within the glenoid cavity was performed by an industrial robot (KR 60-3; KUKA) with 6 degrees of freedom and a position repeatability of 0.06 mm. Forces applied during translation were measured by a force torque sensor (Mini45; ATI Industrial Automation) with an accuracy of 0.25 N, which was placed between the humeri and the robot. For each specimen, an individual 3D coordinate system was established, using a 3D measuring arm (Absolute Arm 8320-7; Hexagon Metrology) with a measurement error <0.05 mm. Anatomic reference points were captured in both the glenoids and the corresponding artificial humerus to build the specimen-specific coordinate system in accordance with each glenoid’s superior-inferior and anterior-posterior axes. A software for robotic joint testing (simVITRO; Cleveland Clinic BioRobotics Lab) was used for translation of the humeral head. Also, the position of the humeral head within the calibrated axes was detected.

### Experiments

Biomechanical testing was performed in 60° of glenohumeral abduction, corresponding to 90° of thoracohumeral abduction in vivo. In midrange motion, the mechanism of concavity-compression is known to be the essential factor for glenohumeral stability.^10,22^ Therefore, the impact of glenoid bone loss followed by bone block application can be observed most accurately in this position.

**Figure 3.**
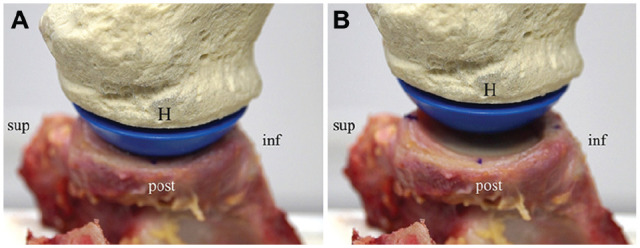
(A) The humeral head (H) is positioned in the starting position within the deepest point of the glenoid cavity. (B) Anteriorly directed force is applied to the humeral head, resulting in anterior translation. The superior (sup), inferior (inf), and posterior (post) orientation of the glenoid-based coordinate system is outlined.

To align the humeral head to the deepest point of the glenoid cavity and to simulate the rotator cuff’s compressive forces, a medially directed compression force of 50 N was exerted to the humeral head. This was based on previous biomechanical studies using similar glenohumeral compression.^10,14,21,22,42,44^ Also, 50 N of compressive force was shown to not cause gross damage to the surrounding tissue during both prolonged compression and anterior dislocation.^21,22^ This position was used as the starting point for humeral head translation. For humeral head translation, an anteriorly directed force was applied to the humerus at a maximum displacement rate of 1 mm/s (Figure 3). Anterior-posterior movement of the humeral head was therefore position-controlled. However, superior-inferior and medial-lateral movement of the humerus was not constrained, resulting in the humeral head seeking the path of least resistance.

Testing was performed in the following testing stages (Figure 1):

1. Intact glenoids
2. 20% aGBL
3. 20% aGBL + flush bone block in level with glenoid
4. 20% aGBL + medialized bone block (–1 mm)
5. 20% aGBL + lateralized bone block (+1 mm)

Between the testing stages, the specimens were carefully moistened with sodium chloride solution spray, so that the articular surfaces did not dry out.

Analogous to previous biomechanical studies,^7,10,22,42^ the stability ratio (SR) was defined as the maximum anterior translational force divided by the constant glenohumeral compression force of 50 N. Multiple comparisons of the SR included comparison of the 20% aGBL stage with every other testing stage, as well as comparison of the flush bone block stage with every other stage.

Before translation, the position of the humeral head was monitored, and the minimum lateral position of the humerus was detected after applying 50 N of compressive force. Since anterior-posterior and superior-inferior humeral movement was not constrained during glenohumeral compression, the medial-lateral starting position of the humeral head within each testing stage was reliably assessed. This also validated the accuracy of bone block positioning. Multiple comparison analyses included comparison of the 20% aGBL stage to flush, medialized, and lateralized bone block positioning, as well as comparison of flush bone block to medialized and lateralized bone blocks.

### Statistical Analysis

A custom-made MATLAB script (R2019a; The MathWorks) was used for signal processing. Statistical analyses of the SR and lateral humeral head deflection at each stage were performed using GraphPad Prism 9 (GraphPad Software). For group comparisons, 1-way repeated-measures analysis of variance and the Sidak post hoc test with a correction for multiple comparisons and a significance level of *P* < .05 were used. The results are presented as mean values, standard deviations, and 95% confidence intervals. Based on pretests and previous publications,^10,21,22^ an effect size (*d_z_*) 1.2 was assumed, resulting in a total sample size of 10 to achieve a statistical power of 0.95.

## Results

### Stability Ratio

**Figure 4.**
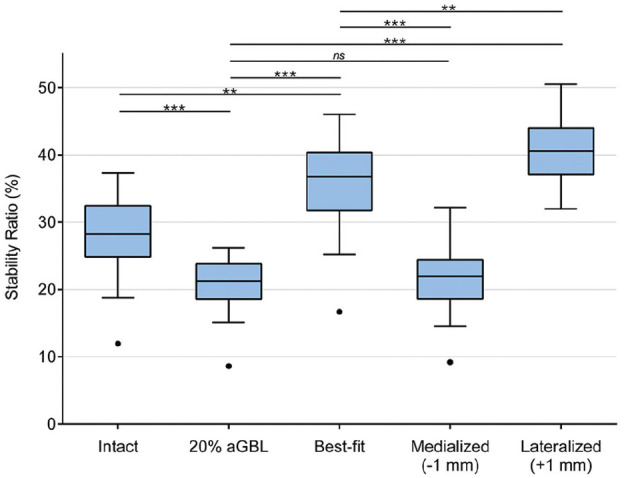
Stability ratio (SR) is dependent on bone block positioning. In 20% anterior glenoid bone loss (aGBL), the SR is significantly lower than that in native joints. With flush and lateralized bone block placements, the SR was significantly increased. With 1-mm medialized bone block positioning, the SR did not differ compared with 20% aGBL. Outliers (•) and levels of significance are outlined: **P < .01; ***P < .001. ns, not significant.

The graft positioning significantly affected the glenohumeral SR (Figure 4). The mean SR in 14 intact glenoids was 27.8% (SD, 6.7%; 95% CI, 23.9%-31.7%), which was significantly reduced by applying 20% aGBL to the specimens (20.6%; SD, 4.7%; 95% CI, 17.9%-23.3%; *P* < .0001).

After reconstructing the defect with the bone block in the flush position, the mean SR was increased to 35.4% (SD, 7.7%; 95% CI, 31%-39.9%). The increase showed statistical significance compared with both the intact glenoids (*P* = .002) and glenoids with 20% aGBL (*P* < .0001).

With a 1-mm medialized bone block position (–1 mm), the mean SR resulted in 21.5% (SD, 5.7%; 95% CI, 8.2%-24.9%). Here, the SR did not differ compared with the specimens with 20% aGBL (*P* = .908) and was significantly lower compared with flush bone block positioning (*P* < .0001).

The 1-mm lateralized bone block position led to a mean SR of 40.8% (SD, 5%; 95% CI, 37.9%-43.7%), which was significantly higher than in 20% aGBL (*P* < .0001) and the flush bone block position (*P* = .003).

### Minimum Lateral Humeral Head Position

**Figure 5.**
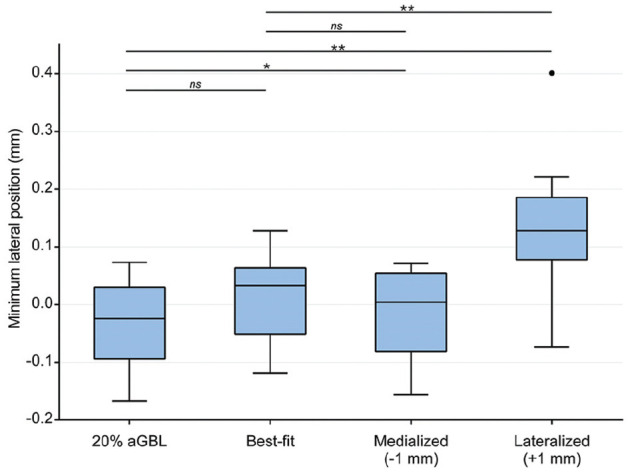
Minimum lateral position of the centered humeral head before anterior translation. One-millimeter lateralized bone block positioning led to a significantly lateralized starting position. One outlier (•) and levels of significance are outlined: *P < .05; **P < .01. aGBL, anterior glenoid bone loss; ns, not significant.

In 20% aGBL compared with flush bone block positioning, the mean minimum lateral position did not significantly differ (–0.035 mm [SD, 0.077 mm; 95% CI, –0.079 to 0.01 mm] vs 0.01 mm [SD, 0.076 mm; 95% CI, –0.034 to 0.053 mm]; *P* = .104) (Figure 5). The comparison of specimens with 20% aGBL to medialized bone block positioning (mean, –0.014 mm; SD, 0.077 mm; 95% CI, –0.058 to 0.031 mm) showed a statistically significant difference (*P* = .034). Between flush and medialized bone block positions, the mean minimum lateral positions did not differ (*P* = .64).

A lateralized bone block position was associated with lateralized starting positions (0.013 mm; SD, 0.118 mm; 95% CI, 0.058-0.031 mm) serving as a control for correct bone block placement. The starting position in lateralized bone blocks was significantly more lateralized than in specimens with 20% aGBL (*P* = .005) and flush bone blocks (*P* = .003).

## Discussion

In this biomechanical study addressing glenoid augmentation in the presence of GBL, the hypothesis that minimal changes in bone block placement relevantly affect osteochondral glenohumeral stability was confirmed. The following main findings can be summarized:

1. Anterior bone block implantation in a flush position restored osteochondral glenohumeral stability and, in this study, increased stability compared with the native, intact glenoids.
2. Bone block medialization of 1 mm led to a significant loss of bony stability compared with a flush bone block position. Furthermore, medialized bone block stability did not significantly differ from glenohumeral stability in the presence of 20% glenoid bone loss.
3. A 1-mm lateralized bone block provided higher bony stability compared with flush bone block positioning. This goes along with a significantly lateralized starting position of the humeral head.

In this study, flush bone block positioning provided an increase in osteochondral stability compared with both the defect stage with 20% aGBL and the initial, native glenoids. The latter was not expected and might be caused by minimal inaccuracy in specimen preparation or bone block positioning. Another explanation is a possible plastic deformation of the chondral glenoid surface during screw fixation of the bone blocks, leading to slightly higher resistance during anterior humeral head translation. Nevertheless, the significantly higher SR proves the positive impact of flush bone blocks on bony glenohumeral stability.

During bony glenoid augmentation in patients with aGBL and anterior glenohumeral instability, the correct bone block positioning is challenging. In the literature, the rates of both bone block malposition and migration differ widely. Graft medialization is associated with persistent anterior shoulder instability. Hovelius et al^12^ described that >80% of patients with graft medialization ≥1 cm after Latarjet procedures experience recurrent shoulder dislocation. In several studies, bone block medialization is defined as a minimum medial displacement ≥5 mm.^1,32,38^ Considering the presented data in this study, a bone block medialization of as much as 1 mm fails to increase osteochondral glenohumeral stability and therefore counteracts the therapeutic intention. Mizuno et al^24^ assessed bone block medialization >1 mm and described rates of 7.4%. Apart from this study, however, we assume the rate of stability-relevant graft medialization is underestimated.

Regarding lateralized bone blocks, many studies have described the association of graft lateralization and the development of osteoarthritis.^18,23,24^ Lalanne et al^18^ found osteoarthritis in 71.4% of patients with lateralized bone blocks after the Latarjet procedure, with a mean follow-up of 22 years. Only 16.7% of patients with nonlateralized bone grafts developed osteoarthritis in the follow-up period. Martins et al^23^ performed finite element models, which showed higher glenohumeral contact pressures causing osteoarthritis, as well as higher glenohumeral stability with a graft lateralization of 3 mm. This falls in line with the presented data in this study, showing the highest amount of glenohumeral stability with a 1-mm lateralized bone block. The lateralized starting position could be one factor for rising glenohumeral contact pressure by increasing distension of the rotator cuff in vivo. These data suggest that even with minimum graft lateralization, an increased risk of osteoarthritis can occur.

To achieve the best possible bone block position, intraoperative placement and fixation, as well as the role of postoperative graft migration, have to be considered. Hovelius et al^12^ described a postoperative graft migration >5 mm in 5% of patients after the Latarjet procedure. In a bony cadaveric model, Youssef et al^45^ analyzed micromotions between bone block and glenoid while performing cyclic load testing with glenohumeral contact pressures up to 170 N. In the absence of soft tissue surrounding, a mean irreversible medialization of 0.91 mm for coracoid grafts and 1.87 mm for scapula spine grafts was detected. Referring these results to the presented data in this study, a relevant, destabilizing graft migration can occur within the postoperative period until bone healing is complete. Do et al^5^ recently analyzed the restoration of a congruent glenoid cavity within 6 months after Latarjet procedure. Here, 8 of 10 initially lateralized bone grafts presented with flush bone block positions 6 months postoperatively. Medialization of an initially flush graft occurred in 1 of 17 patients. Regarding the risk of developing osteoarthritis in lateralized bone blocks,^18,23,24^ Martins et al^23^ found no relevant increase in glenohumeral contact pressure with 1.5-mm lateralized bone blocks. According to our study, preventing even minimal bone block medialization is crucial for bony stability. Thus, considering the capability of restoring a congruent glenoid cavity,^5^ the intentional, slight lateralized graft placement can be discussed. Further studies, however, are required to confirm postoperative, physiological glenoid shaping.

Since recent studies have shown that the loss of anterior glenohumeral stability is mainly caused by the loss of concavity,^26,27,29,41^ it appears desirable to restore concavity during glenoid augmentation. Concave-shaped bone blocks could allow flush intra-articular positioning while reaping the benefits of restrained anterior humeral head translation. The positive effect of concave-shaped grafts has not yet been proven in a clinical setting. Rossi et al^31^ recently compared classic Latarjet procedures with congruent-arc Latarjets, which include rotation of the coracoid tip so that the inferior coracoid aspect mimics anterior glenoid concavity. Here, no differences between the two techniques were found regarding recurrence rates and functional outcome in 145 athletes. Still, modifications of bone blocks restoring anterior glenoid concavity should be assessed in future biomechanical studies.

### Limitations

As a limitation of this study, the high specimen mean age of 79 years, as well as the small sample size of 14 specimens, must be mentioned. Regarding the test setup, the glenohumeral compressive force of 50 N is lower than the physiological, muscular compressive force exerted during shoulder motion.^11,13,30^ The test setup of this study was based on previous studies, as the application of 50 N was shown not to cause damage to the surrounding tissue.

Also, it has to be mentioned that at the bone block edges the glenohumeral contact area was slightly enlarged by adding the 1-mm or 2-mm buildup shells. Since isolated anteriorly directed force was applied to the humeral head, we consider this effect to be negligible as the humeral track during translation was not affected by this.

Regarding the assessment of the minimum lateral starting position, a slight difference between the specimens with 20% aGBL and medialized bone blocks was found, which was not expected before testing. We consider this most probably to be caused by minimal measurement inaccuracy and the small sample size. The lateralized starting position in lateralized bone block placement provides stronger statistical significance.

Finally, soft tissue stabilizing factors were not included in the test setup, as joint capsule, glenohumeral ligaments, labrum, and the rotator cuff have been resected. Thus, the biomechanically stabilizing Latarjet-specific sling effect exerted by the conjoint tendons and the subscapularis muscle was not included either.^9,40,44^ One could suggest that with medialized bone block positioning, glenohumeral stability is still increased due to a larger glenoid surface area resulting in higher capsular and ligamentous tension. This would explain the higher, clinically relevant amount of graft medialization in the literature.^12,32,38^

This study, however, aimed at assessing the osteochondral glenohumeral stability and how this is influenced by bone block positioning. Further studies are supposed to include soft tissue stabilizing factors and rotator cuff loading in order to evaluate global shoulder stability. Also, concave-shaped bone blocks restoring the stabilizing concavity-compression mechanism need to be further evaluated biomechanically.

## Conclusion

In the presence of 20% aGBL, flush bone block placement restores osteochondral glenohumeral stability, while a 1-mm medialized bone block fails to increase osteochondral stability. Bone block lateralization of 1 mm provides higher stability than flush graft positioning, but at the same time, it can lead to humeral head lateralization and an increased risk of developing osteoarthritis.

## Footnotes

§ References 1, 3, 16, 18, 20, 24, 32, 35, 38.
